# Case Report: A research on a case of hereditary spherocytosis with idiopathic pulmonary hemosiderosis

**DOI:** 10.3389/fped.2026.1758140

**Published:** 2026-07-22

**Authors:** Wen xiang Li, Jing Yang, Ting Dong, Deng yan Wu, Li na Ma, Hui min Wu

**Affiliations:** Department of Pediatrics, Lanzhou University Second Hospital, Lanzhou, China

**Keywords:** children, genetic testing, hereditary spherocytosis (HS), pulmonary hemosiderosis, spherocyte

## Abstract

**Background:**

To share the experience of diagnosis and treatment on hereditary spherocytosis (HS) with idiopathic pulmonary hemosiderosis (IPH).

**Methods:**

The clinical data of HS with IPH in a child were retrospectively analyzed based on relevant references.

**Results:**

A 9-year-old girl who had been suffering from anemia for five years received ineffective treatment with iron, folic acid, and vitamin B12 supplements. She had never experienced hemoptysis, shortness of breath, or jaundice. Her hemoglobin (HGB) level was 71 g/L, and chest computed tomography (CT) revealed slight flocculent exudation. Hemosiderin-laden macrophages (HLMs) were detected in her bronchoalveolar lavage fluid (BALF).After the possibility of autoimmune hemolytic diseases was ruled out, the patient was diagnosed with IPH and treated with glucocorticoid medication (oral prednisone). Her HGB level gradually recovered but decreased again after 4–5 months of treatment, which was not related to the reduction of glucocorticoid (prednisone). At the same time, she exhibited icteric sclera, spherocytes (at 24%) in the peripheral blood smear, and a positive erythrocyte fragility test result. Genetic testing revealed heterozygous alterations in her ANK1 gene (1) and PKLR gene (2), as well as two heterozygous alterations in her SPTB gene (3). Finally, the patient was diagnosed with HS and IPH. Conclusion: IPH should be considered in children with chronic anemia that does not respond to treatment with iron and vitamin supplements.

**Conclusions:**

Furthermore, medical history, especially test results showing mild abnormalities, is of great importance for the accurate diagnosis of hereditary diseases.

## Introduction

HS is a hemolytic disease caused by congenital erythrocyte membrane defects, which is mainly characterized by varying degrees of anemia, recurrent jaundice, splenomegaly, spherocytosis, and increased osmotic fragility of erythrocytes. As the most common type of hereditary erythrocyte membrane defect disease, HS is not rare in China; however, its clinical detection rate is significantly lower than its incidence rate (1). IPH is a rare disease that causes recurrent episodes of diffuse alveolar hemorrhage (DAH) in children, especially those under the age of 10. It is characterized by hemoptysis, iron-deficiency anemia (IDA), and pulmonary infiltration on chest imaging (2–4), and even chest distress, shortness of breath or dyspnea can be identified by pulmonary hemosiderin-laden macrophages in BALF, sputum, or gastric juice (2, 5). However, children with IPH often do not exhibit any of the above typical clinical manifestations, but instead present only with symptoms such as chronic anemia or recurrent cough. Due to its low incidence rate, misdiagnosis and mistreatment are common in clinical settings. The following is a medical report for a child with HS and IPH admitted to our hospital.

## Case report

A 9-year-old girl was admitted to the hematology department of our hospital due to anemia. The patient, a 9-year-old girl, was admitted to our hospital in June 2019 due to a 5-year history of anemia and intermittent fever and cough for 14 days. Five years ago, the patient was diagnosed with anemia at a local hospital due to epistaxis and received unspecified symptomatic treatment, which had poor efficacy. In March 2019, the patient underwent bone marrow puncture, which revealed hyperplastic anemia. The erythrocyte osmotic fragility test (EOFT) showed 4.8 g/L (in primary hemolysis) (reference value: 3.8–4.6 g/L) and 3.6 g/L (in complete hemolysis) (reference value: 2.8–3.2 g/L). Ham test, sucrose hemolysis test, qualitative urine hemosiderin test, methemoglobin reduction test (MHB-RT), and Heinz body formation test all yielded negative results. Autoimmune antibody testing was also negative. Granulocyte and erythrocyte CD55 and CD59 tests were negative, and serum bilirubin levels were within normal limits. After the possibility of hemolytic anemia and hemorrhagic anemia was ruled out, Iron Proteinsuccinylate Oral Solution, folic acid, and vitamin B12 were prescribed as discharge medications. However, there was no significant increase in her hemoglobin after a three-month treatment. Before her admission, the child had a fever with a peak temperature of 39.3 °C that lasted for 14 days, accompanied by paroxysmal cough and expectoration, without hemoptysis or dyspnea. The child was then hospitalized due to bronchopneumonia and anemia. The child's parents were divorced; her father is healthy, while her mother's physical condition remains unknown.

**Figure 1:**
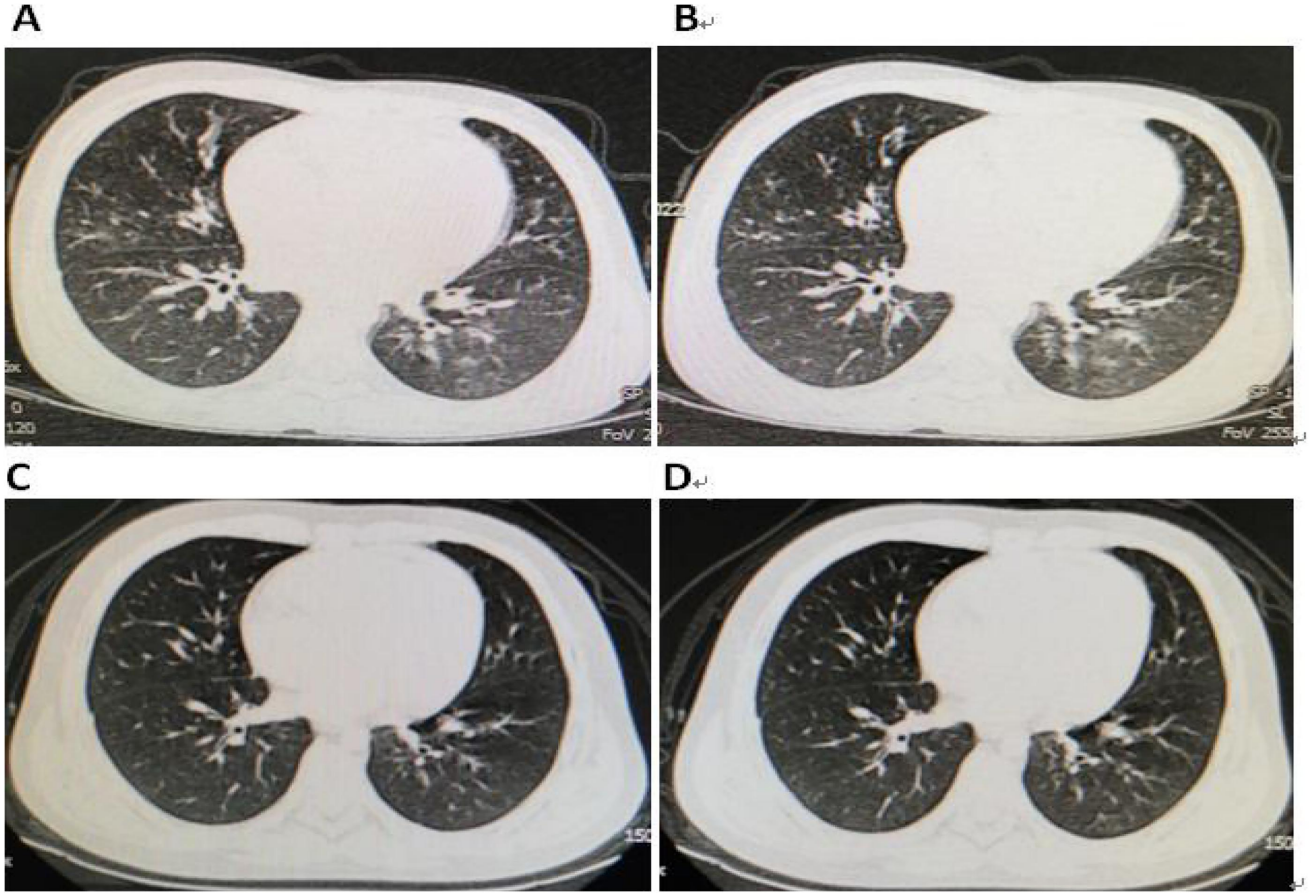
**(A,B)** nodular, small patchy and flocculent increased-density shadows observed in June 2019. **(C,D)** Exudation from both lungs was adsorbed and improved in Nov. 2019.

Diagnosis on admission: The child was moderately nourished and presented with signs of moderate anemia. There was no palpable enlargement of superficial lymph nodes or jaundice. Breath sounds were rough in both lungs, with fixed moist rales heard at the base of the left lung. The liver was normal in size and not palpable under the ribs, while the spleen was located 2.5 cm below the left costal margin and was of medium hardness. Laboratory findings are summarized in Tables 1, 2. Peripheral blood smear showed no abnormalities; serum bilirubin levels were normal; mycoplasma pneumoniae antibody titer was 1:640. Chest CT revealed a small amount of patchy opacity in both lungs (see Figures 1A,B). Bone marrow puncture indicated hyperplastic anemia. No esophageal or gastric mucosal erosion or bleeding was observed during electronic gastroscopy. No active intrabronchial hemorrhage was found during electronic bronchoscopy. Hemosiderin-laden macrophages were detected in the BALF collected, and the result was positive. According to the patient's clinical manifestations and examination results, she was diagnosed with mycoplasma pneumoniae pneumonia (MPP) and IPH. The patient was then treated with azithromycin sequential therapy and oral prednisone (2 mg/kg·d). When her cough improved and her body temperature returned to normal, she was discharged from the hospital with medication and underwent regular outpatient follow-up visits. See Tables 1, 2 for relevant examination results and oral prednisone doses within six months after discharge. In January 2020, the child was diagnosed with jaundice; spherocytes accounted for 24% in the peripheral blood smear, and erythrocyte fragility test results were 5.5 g/L before incubation (reference value: 4–4.5 g/L) and 6.26 g/L after incubation (reference value: 4.65–5.9 g/L). The Ham test, sucrose hemolysis test, qualitative test of urine hemosiderin, methemoglobin reduction test (MHB-RT), and Heinz body formation test all yielded negative results. Autoimmune antibody testing also yielded a negative result. Plasma free hemoglobin (PFH) was 97 mg/L (reference value: <40 mg/L); Hemosiderin-laden macrophages were detected in her BALF, resulting in a positive result; and bone marrow aspiration indicates hyperplasia of erythroid series. Genetic testing for hereditary blood and immune system diseases in the child and her biological father revealed that: (1) there was a heterozygous alteration in her ANK1 gene, which was inherited from her father; (2) there was a heterozygous alteration in her PKLR gene; and (3) there were two heterozygous alterations in her SPTB gene. In addition, examinations of the patient's father indicated that he had normal blood routine, bilirubin levels, and peripheral blood smear, without splenomegaly. When asked about her mother's physical condition, her father recalled that her mother had a history of intermittent pallor, but she could not be contacted so far. Thus, the patient was diagnosed with HS and IPH. Since the patient's hemoglobin fluctuated between 90 and 110 g/L, with her spleen located 2.5 cm below her ribs, her father was informed of her illness. Considering that the patient's hemoglobin is greater than 90 g/L and she is relatively young, her father chose hormone therapy. Glucocorticoids were used and the dosage was gradually reduced. Splenectomy was not performed for the time being.

**Table 1:** Laboratory finding.

Date	WBC ( × 10⁹/L)	RBC ( × 10¹²/L)	Hb (g/L)	MCV (fL)	MCH (pg)	MCHC (g/L)	RDW-SD (%)	RDW-CV (%)	Ret absolute value ( × 10¹²/L)	Spherocytes in peripheral blood smear (%)	Dose of oral glucocorticoids (mg)
March 26, 2019	4.2	2.81	77	86.5	27.4	317	72	23.4	0.085	Not found	–
June 1, 2019	4.1	1.9	71	88.9	37.4	420	56	26.1	–	Not found	–
June 3, 2019	4.77	2.23	72	88.3	32.3	365	67	24.7	0.067	Not found	–
June 10, 2019	7.4	2.37	78	92	32.9	358	80	27.6	0.13	Not found	25 qd
July 10, 2019	8	3.96	115	93.4	29	311	66	20.2	–	Not found	12.5 qd
Aug. 14, 2019	5.2	3.69	109	92	30	326	70	22.3	–	–	10 qd
Sept. 11, 2019	6.3	3.7	111	89.5	30	335	66	21.6	–	–	7.5 qd
Oct. 9, 2019	5.7	3.34	102	91.3	29.7	326	68	23	–	–	10 qd
Nov. 13, 2019	5.6	3.33	100	84.7	30	355	57	20.7	–	–	10 qd
Dec. 18, 2019	5	3.1	90	95.5	29	304	78	24.5	–	28	10 qd
Jan. 14, 2020	6.8	3.45	100	88.1	29	329	63	20.6	0.112	26	10 qd
Jan. 19, 2020	6.29	3.29	97	91.5	29.5	322	66	21.7	0.125	24	10 qd

**Table 2:** Laboratory finding.

Date	TBIL (umol/L)	IBIL (umol/L)	Spleen	Proportion of red bone marrow	Indication from bone marrow test	Jaundice	Time of primary hemolysis	Time of complete hemolysis	G6PD	Coombs test	Serum Fe (ng/ml)	Folic acid (mmol/l)	Vitamin B 12 (pmol/l)
March 26, 2019	24.4	22.8	Enlarged	47	Hyperplastic anemia	No	4.8	3.6	Negative	Negative	Normal	Normal	1227
June 10, 2019	24	20.4	Enlarged	28	Hyperplastic anemia	No	–	–	–	–`	Normal	>20	>2000
Dec. 18, 2019	73.3	48.6	Enlarged	–	–	Yes	–	–	–	–	–	–	–
Jan. 19, 2020	46.2	34.7	Enlarged	58	HS	Yes	5.5	6.25	Negative	Negative	Normal	Normal	1044

## Discussion

The patient had chronic anemia that did not respond to treatment with iron, folic acid, and vitamin B12 supplements, with the possibility of active hemorrhage and autoimmune hemolysis ruled out. Her chest computed tomography (CT) during hospitalization at the hematology department of our hospital revealed small patchy and flocculent exudates. With the consent of her parent, hemosiderin-laden macrophages were detected in the bronchoalveolar lavage fluid (BALF), and the result was positive; therefore, the patient was diagnosed with idiopathic pulmonary hemosiderosis (IPH). The patient was treated with oral prednisone, and her HGB level significantly increased, with the flocculent exudation on chest CT markedly improved (see Figures 1C,D). However, the HGB level decreased again after 4–5 months of treatment, which was not related to the reduction of glucocorticoid (prednisone) dosage. At the same time, she exhibited mild jaundice, slightly elevated serum bilirubin (mainly indirect bilirubin), and spherocytes accounted for 28% in the peripheral blood smear. After relevant examinations and genetic screening, the child was diagnosed with hereditary spherocytosis (HS) in addition to IPH. Both IPH and HS are rare conditions, and their coexistence is even rarer. Regarding the patient's previous physical condition, a mild abnormality in EOFT was detected three months before the diagnosis of IPH. At that time, the possibility of HS was dismissed because the child's serum bilirubin level was normal, and no spherocytes were found in the peripheral blood smear or bone marrow test. However, as the hemoglobin levels recovered, spherocytes increased and signs of hemolysis appeared, leading to the diagnosis of HS. In treating this patient, we gained valuable experience and learned important lessons. Experience: IPH should be considered in children with chronic anemia unresponsive to standard treatment, especially those with atypical clinical manifestations. In addition to chronic anemia, this child had no symptoms such as chronic cough, hemoptysis, or shortness of breath. Her chest CT revealed atypical alveolar hemorrhage, which made it easy to lead to misdiagnosis. In this case, the disease can be identified by the presence of pulmonary hemosiderin-laden macrophages in BALF, sputum, or gastric juice. Lessons learned: 1. A detailed family medical history should be obtained. In this case, the patient's parents were divorced, and her father had no history of anemia or hyperbilirubinemia. We were unable to obtain a detailed medical history from her mother, so we may have overlooked the possibility of a genetic cause. 2. Do not ignore any abnormal examination results: We overlooked the mild abnormalities in the EOFT, which caused us to fail to consider the possibility of hereditary spherocytosis (HS) at an early stage.

After consulting references on HS and IPH, we came to the following conclusions.1.Diagnosis of HS requires consideration of the following seven aspects: Clinical manifestations of chronic hemolytic anemia and experimental evidence of hemolysis are essential. A positive family history is strong evidence in diagnosis, but its absence does not negate the diagnosis. The existence of spherocytes in peripheral blood (PB) should be considered, although spherocyte counts should not be deemed a necessary condition for diagnosis. Therefore, when spherocytes account for <10%, the diagnosis cannot be easily dismissed. The erythrocyte fragility test is an important diagnostic index. The possibility of other hemolytic diseases elevating spherocyte counts and increasing the osmotic fragility of erythrocytes should be ruled out (6). Some reported comprehensive analyses indicate that RBC parameters in the blood routine are helpful in providing a diagnostic basis for HS (7). With MCV < 80FL, MCHC > 354 g/L and RDW > 14%, it can be diagnosed as HS sensitivity at 63% and specificity at 100%. At present, five kinds of erythrocyte membrane protein (EMP) coding genes have been found to be associated with HS. They are ANK1, SPTA1, SPTB, SLC4A1, and EPB42 genes, which code for ankyrin, α-spectrin, β-spectrin, band 3 protein, and protein 4.2, respectively. Among these, ANK1 and SPTB are the most common pathogenic genes for HS in Asians (8). 2. For IPH, there are mainly six diagnostic criteria: recurrent cough and shortness of breath, sometimes accompanied by hemoptysis; microcytic hypochromic anemia of unknown origin; diffuse pulmonary infiltration and pulmonary interstitial changes shown on chest radiography or CT; hemosiderin-laden macrophages observed in sputum, gastric juice, or BALF. The sensitivity for detecting hemosiderin-laden macrophages in gastric fluid is 30%, while the sensitivity in BALF is 92% (8, 9). Hemosiderosis and pulmonary fibrosis to varying degrees can be observed in a lung biopsy (the gold standard for diagnosing IPH), which is rarely adopted domestically (8). Excluding other causes of pulmonary hemorrhage, such as systemic lupus erythematosus (SLE), tuberculosis (TB), bronchiectasis, recurrent bronchopneumonia, bronchial foreign bodies, and vascular malformations, most child patients do not manifest typical specific symptoms, but almost every child patient has anemia (10, 11). Glucocorticoid is a first-line drug for IPH. It can shorten the duration of pulmonary hemorrhage and reduce the disease course, thereby lowering the incidence and fatality rates. In addition, splenectomy is an effective treatment for HS.

In this case, no spherocytes were found in the child patient during her early-stage treatment, and this may be related to the following factors: 1. Not all HS patients have significantly increased spherical erythrocytes, so an elevated erythrocyte count is not a prerequisite for the diagnosis of HS. 2. Recurrent alveolar hemorrhage reduces the material available for erythrocyte synthesis and affects erythrocyte morphology and size. 3. Glucocorticoids may affect the stability of the erythrocyte membrane, leading to the formation of more spherocytes. 4. IPH may influence the formation of spherocytes in this HS child patient, which requires further research and discussion.

The clinical diagnosis rate of HS is low, and the diagnosis of HS complicated with other diseases is even more challenging. Therefore, for clinical cases, it is important to obtain a detailed family and personal medical history, and to pay close attention to any symptoms and abnormal examination findings in order to ensure early diagnosis and timely treatment.

## Data Availability

The original contributions presented in the study are included in the article/Supplementary Material, further inquiries can be directed to the corresponding author.
